# A new attempt to remove toluene using nickel–iron bimetallic particle electrode reactor

**DOI:** 10.1038/s41598-024-60956-0

**Published:** 2024-05-02

**Authors:** Siwen Li, Bo Jiang, Gen Liu, Chunyan Shi, Hongbin Yu, Yingzi Lin

**Affiliations:** 1 School of Environment, Northeast Normal University, Changchun, 130117 China; 2 Jilin Research and Design Institute of Building Science (Jilin Province Construction Engineering Quality Test Center), Changchun, 130011 China; 3 School of Municipal & Environmental Engineering, Jilin Jianzhu University, Changchun, 130118 China; 4 The University of Kitakyushu, 1-1 Hibikino Wakamatsuku Kitakyushu, Fukuoka, Japan

**Keywords:** Advanced oxidation processes, Bimetallic regulation, Air purification, Particle electrode wet state, Environmental sciences, Engineering, Materials science

## Abstract

A new attempt of removing toluene waste gas using a three-dimensional electrode reaction device with nickel–iron bimetallic particle electrode is presented in this paper. The particle electrode was prepared by a simple liquid phase reduction method. Through bimetal modification, the particle electrode mass transfer rate is increased to 1.29 times, and the degradation efficiency of the reactor is increased by nearly 40%, which makes it possible to remove toluene waste gas by other electrochemical methods in addition to plasma method. The removal efficiency of the particle electrode can be stabilized at more than 80% after 5 cycles (50 h). At the same time, the relationship between independent working parameters and dependent variables is analyzed using the central composite design, and the operating parameters are optimized. Based on this study, the removal mechanism and possible degradation pathway of toluene were investigated. This study provides a supplement to the possibility and theoretical basis of new technology application for electrocatalytic oxidation removal of VOCs.

## Introduction

Volatile organic compounds (VOCs) not only leads to direct air pollution, but also is a precursor of PM2.5 and ozone^1^. The coordinated control of PM2.5 and ozone can be effectively strengthened by managing VOCs, which is crucial to achieving synergies of carbon reduction and promoting continuous improvement of ecological quality^2^. In addition, according to statistics, people in modern society have more than 80% of the time in indoor activities. VOCs released from building materials and furniture lead to relatively high concentrations of indoor VOCs, which also poses a great threat to human health^3^.

Controlling VOCs technologies include physical, chemical, and biological technologies such as adsorption, incineration, catalytic oxidation, and biodegradation^4–7^. However, these technologies still have some limitations in their wide application. For example, adsorption technology only transfers pollutants rather than effective decomposition and removal, it may bring secondary pollution; incineration technology has low cost, but only suitable for high concentration volatile organic compound treatment; photocatalytic biotreatment technology can react at room temperature, but the reaction rate is slow, and the treatment effect of low concentration discharge is poor ^8–11^. Based on the disadvantages of some current treatment technologies, it is of great significance to develop clean and efficient treatment technologies for the removal of volatile organic compounds^12^.

Advanced oxidation technology is one of the most promising technologies for removing volatile organic compounds^13^. VOCs can be oxidized on the catalyst, and the reaction temperature is much lower than the temperature of the thermal oxidation treatment process or the combustion process. Not only can save energy, get the product is less toxic, even non-toxic^14–23^. Among them, the three-dimensional electrode electrocatalytic oxidation technology has been favored by scholars because of its clean, high efficiency and environmentally friendly characteristics, and has gradually become a hot research direction in recent years. At present, in addition to the plasma method, there are very few studies involving the removal of gaseous volatile organic compounds by electrocatalytic oxidation^24^. Such as a novel UV-assisted PEC-MFC system^24^ and a semi-solid gel-induced triphase interface assembly with membrane-divided electrochemical half-cell assembly^24^ were applied in VOCs removal.

Limitations of removal of volatile organic compounds based on electrocatalysis (such as high energy consumption and cost, certain safety hazards when handling flammable and explosive exhaust gases, etc.), in this study, a three-dimensional electrode reaction device was constructed with self-made GAC@Ni/Fe particle electrode. Toluene waste gas was selected as the model pollutant for experimental study. First, the removal of toluene has a research basis ^25–27^ toluene has a relatively low octanol–water distribution coefficient and a higher Henry's law constant^28,29^, so this study is also widely applicable to a large number of other gaseous VOCs.

In this study, nickel–iron bimetal was used to modify and control the surface of conventional particle electrodes. Strengthening the efficiency of the traditional granular activated carbon electrode to realize the effective removal of VOCs by the three-dimensional electrode reaction device, ecomposing gaseous harmful substances into small molecule pollution-free products, supplementing the research basis of three-dimensional electrode electrocatalytic oxidation technology in the field of VOCs removal, it also provides a new technical research direction for effective VOCs removal in the future.

## Materials and methods

### Chemicals

C_7_H_8_·(AR),·C_2_H_6_O·(AR), FeSO_4_·7H_2_O·(AR), NiCl_2_·6H_2_O·(AR), and Na_2_SO_4_·(AR), were purchased from Sinopharm Chemical Reagent Co., Ltd. NaBH_4_ (AR) was purchased from Shandong Asia Chemical Industry Co., Ltd. H_2_SO_4_·(AR) was purchased from Beijing Chemical Plant. Pure chemicals and water were used.

### Preparation of the particle electrodes

The surface of granular activated carbon was soaked with 0.05 mol/L dilute sulfuric acid, then cleaned with ethanol solution and distilled water respectively, and dried in a constant temperature drying oven for reserve use. 4.5 g of NiCl_2_·6H_2_O and FeSO_4_·7H_2_O solid powder with molar weight Ni:Fe = 1:1 were weighed and dissolved in 100 mL distilled water, then the mixture was transferred to 500 mL three-neck flask, 10 g of pre-treated granular activated carbon was added, and the stirring device was started. Under the protection of nitrogen, the newly configured NaBH_4_ solution was added to the three-neck flask at a constant rate of 1 drop per second. After full reaction for 30 min, the solution was rapidly filtered, and then dried in a constant temperature drying oven at 105 °C for future use^30^.

### Material characterization

Scanning electron microscope (S-4800) and transmission electron microscope (JEOL2100) are used to observe the morphology and elemental composition before and after the preparation of particle electrodes. Fourier transform infrared spectrometer (Spectrum GX) analyzed the change of surface functional groups after particle electrodes. The crystal structure of the particle electrode was analyzed by an X-ray diffractometer (X Pert-Pro MPD). X-ray photoelectron spectroscopy (Thermo250XI) analyzes the bimetallic valence states of the particle electrode surface. An electronic paramagnetic resonance spectrometer (Bruker EMXnano desktop EPR spectrometer) was measured at room temperature. Measure the contact Angle by using the contact Angle meter. The Princeton (P4000A) electrochemical workbench measures the electrocatalytic effect of the particle electrodes. Agilent (7890B-7000C) analyzed possible degradation products.

### Degradation experiments

All removal experiments were carried out in a self-made reactor. The experimental reaction apparatus is shown in Fig. S1 (supplementary materials: Schematic diagram of the experimental setup). The average diameter of bimetallic particle electrode is 2 mm, the initial electrolyte is 0.01mol/L sodium sulfate solution, and the initial voltage is 15 V. The simulated exhaust gas enters the lower end of the three-dimensional electrode reaction device 6, and is discharged from the upper part of the 6 after being treated by the gas–solid-liquid reaction zone.

The electrocatalytic degradation experiment was carried out after the particle electrode reached adsorption equilibrium. The factors influencing the degradation were investigated. Some of the key factors were tank voltage, particle electrode dosage, gas flow rate and electrolyte concentration. In the quenching experiment, tert-butanol, L-histidine and p-benzoquinone were used as quenching agents to determine the main active species. The recycling experiment takes every ten hours as a cycle to investigate the removal effect of the particle electrode on the target pollutants. All experiments were repeated 3 times, and the mean and standard deviation were calculated.

### Analytical methods

The inlet and outlet gas samples were collected every 10 min with a gas sampling bag. The experiment lasted for 60 min. The concentration of gaseous toluene was detected by a gas chromatograph. In this study, the proportion of gaseous toluene reduced from the upper space of the reactor was defined as "removal ratio (Rt)", and the Eqs. (1) was used to calculate:

1 $$ {\text{R}}_{{\text{t}}} = \, \left( {{\text{C}}_{{{\text{in}}}} - {\text{ C}}_{{{\text{out}}}} } \right)/{\text{C}}_{{{\text{in}}}} \times { 1}00 \, \left( \% \right) $$

C_in_ and C_out_ are the concentrations of imported and exported gaseous pollutants at the sampling time, respectively^31^.

Self-made bimetallic particle electrode, platinum sheet electrode and Ag/AgCl electrode were used as counter and reference electrodes. The CV and LSV data were measured using an electrochemical workstation with a scan rate of 0.01—0.2 V s^-1^. The Nyquist map was obtained by electrochemical impedance spectroscopy with a frequency range from 0.01 Hz to 100 kHz. Pure nitrogen was pumped into the solution for 10 min before testing. Tafel formula can well reflect the electron transport kinetics of different electrodes, its expression is: η = a + b × log|i|. Where: η: overpotential (V); i: Current density (A/cm^2^); a, b: Tafel constant. All curves except CV were not infrared calibrated, and all potentials were corrected by the reversible hydrogen electrode (RHE) by Nernst correlation method (E_RHE_ = E(Ag/AgCl) + 0.059 pH + 0.197)^32^.

## Results and discussion

### Structural characterization of particle electrodes

**Figure 1:**
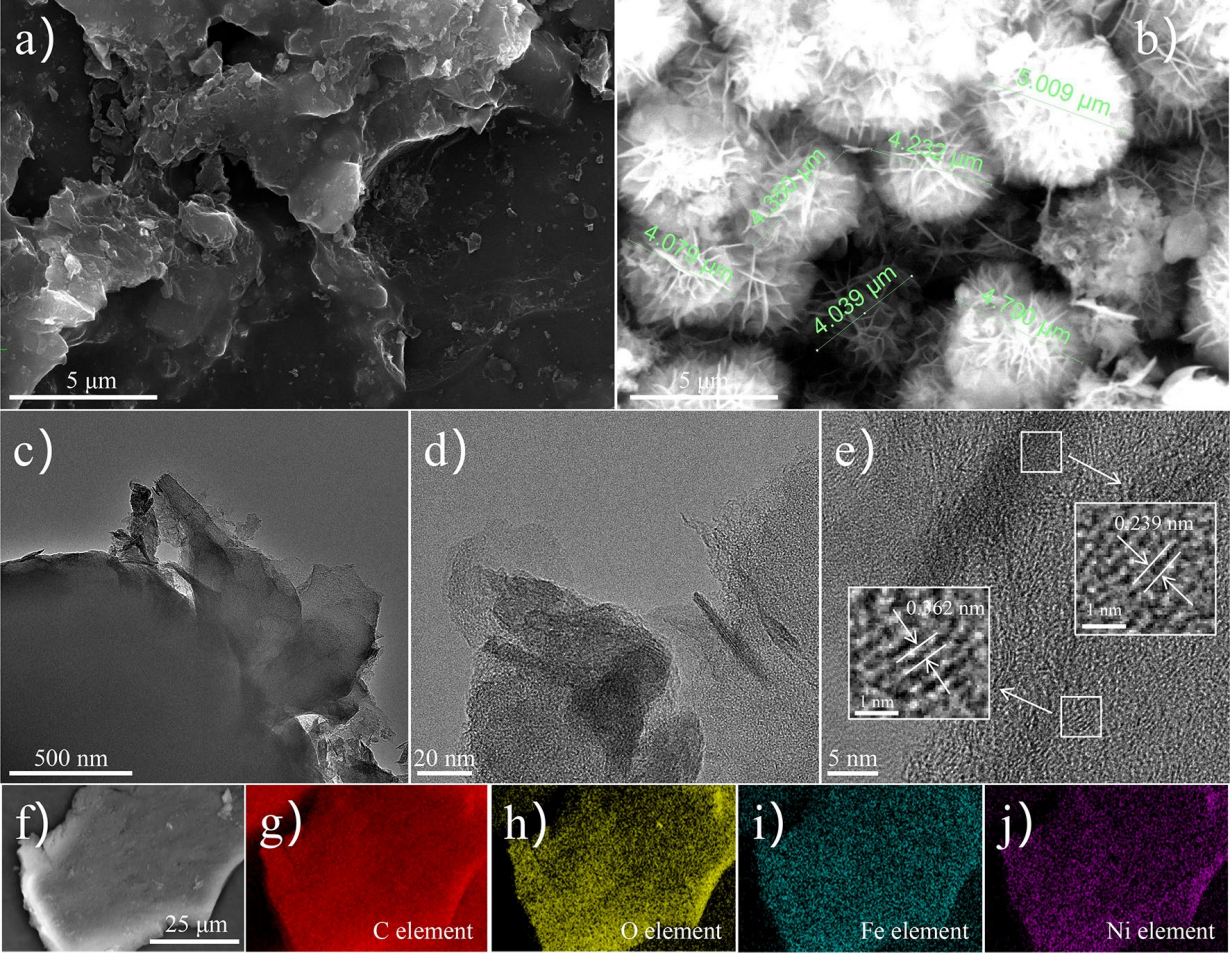
SEM images of GAC (**a**) and GAC@Ni/Fe (**b**); TEM images of GAC@Ni/Fe (**c**, **d**); HRTEM image (**e**) and elemental mappings (**f**–**j**) of GAC@Ni/Fe.

The morphology and microstructure of bimetallic particle electrodes were studied by scanning electron microscopy and transmission electron microscopy. As shown in Fig. 1a, the surface of the granular activated carbon without metal loading is clear and smooth; After loading the metal, as shown in Fig. 1b, the surface presents spherical petal-like particles superimposed with an average size of about 4.5 microns. Figure 1c and Fig. 1d are TEM images of particle electrodes, with clear and smooth boundaries and transparent state, indicating the ultrathin characteristics of petals^33^. Figure 1e is the HRTEM diagram of the GAC@Ni/Fe particle electrode. The visible lattice fringes also correspond to the oxide crystal faces of iron and nickel, respectively. The element mapping (Fig. 1f–j) further shows the uniform load of nickel and iron metal elements, and the presence of oxygen elements also proves the formation of metal oxides, which are partly caused by the oxidation of particles during washing, drying and testing. These results provide further evidence for the successful preparation of bimetallic particle electrodes.

**Figure 2:**
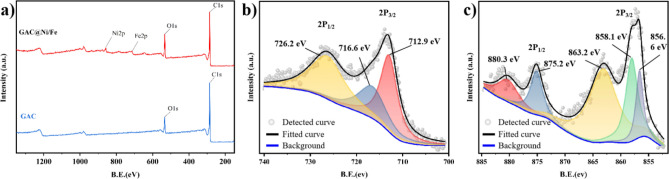
XPS total spectrum of the GAC@Ni/Fe and GAC (**a**); High-resolution XPS spectra in the regions of Fe 2p (**b**) and Ni 2p (**c**) for the GAC@Ni/Fe.

In order to further understand the surface chemical state of the particle electrode, the surface of the bimetallic particle electrode was analyzed by XPS method. As shown in Fig. 2a, Ni and Fe elements were added to the surface of the particle electrode after loading the bimetal. Figure 2b is a spectrogram of Fe 2p with peaks representing iron oxides at binding energies of 712.9 eV, 716.6 eV, and 726.2 ev^34^. Figure 2c is the spectral diagram of Ni 2p. The binding energies of 856.6 eV, 858.1 eV, 863.2 eV, 875.2 eV and 880.3 eV represent the characteristic and satellite peaks of nickel 2p_3/2_ and 2p_1/2_ oxides, respectively^35^. These results show that bimetallic iron and nickel are successfully loaded and exist in the form of oxides.

### Electrochemical characterization of particle electrodes

#### Analysis of mass transfer rate and electrochemical active surface area

In this study, the scan rates were controlled as follows: 0.2 V/s, 0.1 V/s, 0.05 V/s and 0.01 V/s, respectively. The cyclic voltammetry curves (CV) of GAC, GAC@Ni, GAC@Fe and GAC@Ni/Fe particle electrodes and the cyclic voltammetry curves (CV) of GAC@Ni/Fe particle electrodes loaded with different proportions of Ni and Fe metal were tested respectively. Good linear relationship between the peak oxidation current (I_P_) and the square root (V^1/2^) of the scan rate: I_P_ = kV^1/2^. Through experiments, we found that the mass transfer rate of GAC particle electrode k_GAC_ = 0.3221, when loaded with single metal, the mass transfer rates of GAC@Ni and GAC@Fe are increased to k_GAC@Ni_ = 0.3403 and k_GAC@Fe_ = 0.3869, respectively. When loaded with bimetal simultaneously, the mass transfer rate of GAC@Ni/Fe particle electrode is 1.29 times that of pure GAC particle electrode, k_GAC@Ni/Fe_ = 0.4166. This shows that different metal loads have a good effect on improving the mass transfer rate of conventional GAC particle electrodes. The results of which are shown in Fig. S2 and Table S1. At the same time, we explored the improvement of the electrochemical active surface area of the particle electrode by the ratio of Ni and Fe metal loading, and the experimental results were shown in Fig. S3. When the bimetal loading ratio is 1:1, the electrochemical performance of the particle electrode is the best.

#### Electrocatalytic activity and the kinetics of electron transport

**Figure 3:**
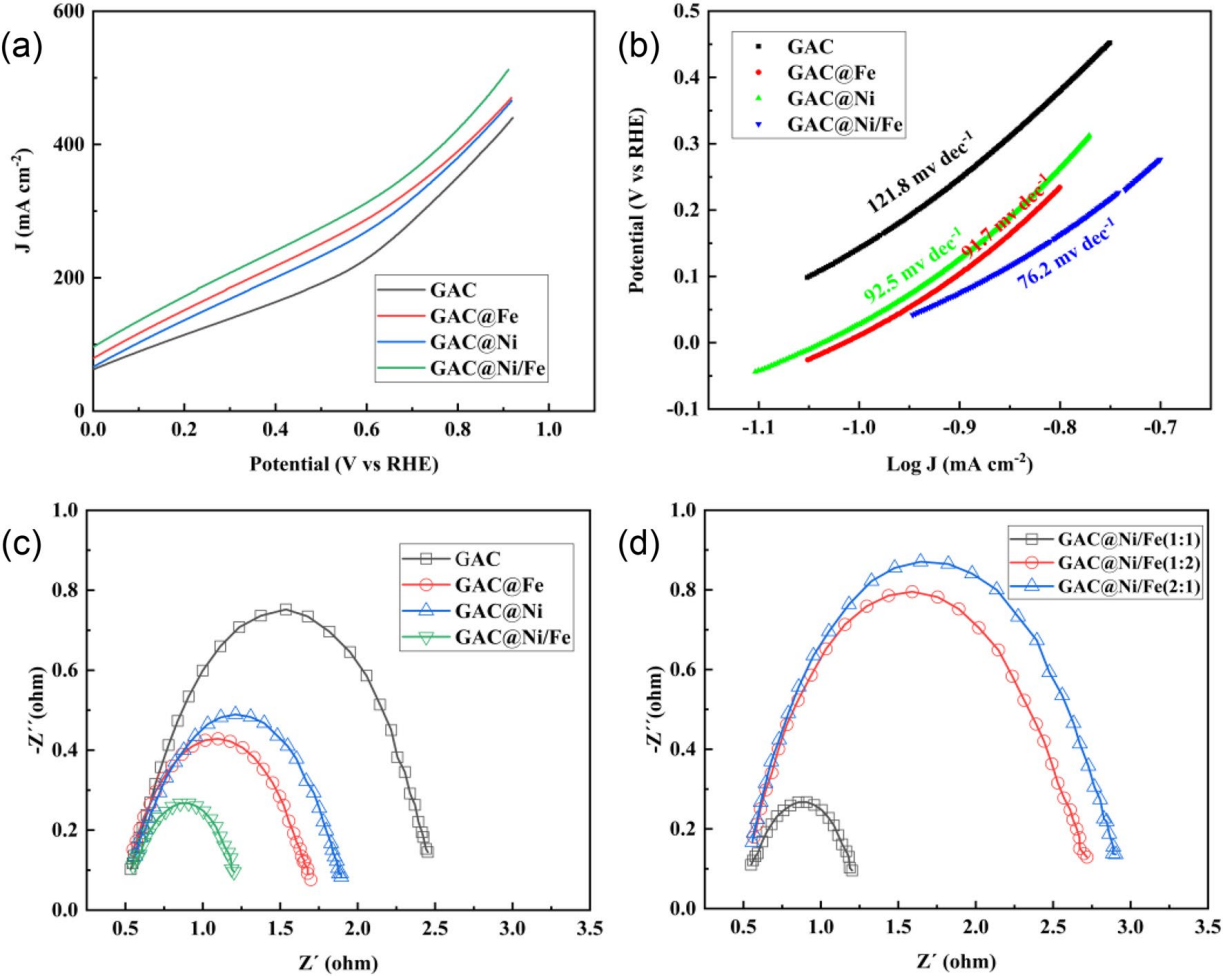
Linear voltammetry (LSV) curves of different particle electrodes (**a**); Tafel curves for different particle electrodes (**b**); Ac impedance diagram of different particle electrodes (**c**); Ac impedance diagram with different metal loads (**d**).

In this study, linear voltammetry curve (LSV) was used to evaluate the electrocatalytic activity of particle electrodes. As can be seen from Fig. 3a, linear voltammetry curves (LSV) of GAC, GAC@Ni, GAC@Fe and GAC@Ni/Fe electrodes with different particle sizes show a similar trend. When the current value is the same, the lower the potential, the higher the electrocatalytic activity of the tested sample^36^. As shown in the figure, when the current is 200 mA, GAC@Ni/Fe has a potential of 0.28 V, while GAC@Ni and GAC@Fe loaded with single metal are only 0.442 V and 0.408 V respectively, which is lower than 0.528 V of GAC. Thus, we can obtain, the GAC@Ni/Fe particle electrode supported by bimetal has higher catalytic activity.

Comparison of linear voltammetry curves of different particle electrodes (LSV), when the current is 0–100 mA, The electrocatalytic activity of GAC, GAC@Ni and GAC@Fe particle electrodes is basically consistent. This shows that the single metal loading can not effectively improve the electrocatalytic effect of the particle electrode under the condition of low current. With the increase of current, the potential growth rate of GAC, GAC@Ni, GAC@Fe and GAC@Ni/Fe particle electrodes decreases gradually. Therefore, it is also shown from the side that the advantages of electrocatalytic activity of the bimetallic particle electrode in this study become more obvious with the increase of current. By comparing the linear voltammetry (LSV) curves of GAC@Ni/Fe particle electrodes with different metal loading ratios, we can also find that the catalytic activity is best when the ratio is 1:1, as shown in Fig. S4.

Tafel curves can well reflect the electron transport dynamics of different electrodes. We analyzed the fitting data of the linear region of Tafel curves of several particle electrodes, as shown in Fig. 3b. It can be seen from the figure that GAC@Ni/Fe particle electrode has the lowest Tafel slope. It shows that it can achieve faster electron transport dynamic rate^37,38^.

In conclusion, the linear voltammetry curve (LSV) and Tafel curve (Tafel) of GAC, GAC@Ni, GAC@Fe and GAC@Ni/Fe particle electrodes were tested in this study, as well as the linear fitting of Ig[current density (A/cm^2^)] and potential [V] in the linear interval of TAFEL. The bimetallized particle electrode GAC@Ni/Fe has a faster kinetic rate of electron transport. The electrochemical performance is the best when the metal loading ratio is 1:1.

### EIS analysis of particle electrode

Electrochemical impedance spectroscopy (EIS) is a simple and powerful chemical analysis technique for measuring electrical conductivity. In order to study the electrochemical response of bimetallic particle electrode, EIS test was carried out when AC disturbance amplitude was 100 kHz–0.01 Hz. EIS studied the charge transfer energy of the particle electrode^39^. Nyquist diagram of GAC, GAC@Ni, GAC@Fe and GAC@Ni/Fe particle electrodes is shown in Fig. 3c. Each image is semi-circular in the high frequency region and linear in the low frequency region. The diameter of the semicircle corresponds to the charge transfer resistance (Rct) of the electrode/electrolyte interface REDOX reaction. It is generally believed that the larger the semicircle, the greater the charge transfer resistance, the steeper the slope, and the lower the ion diffusion rate. Nyquist plots of the three samples in Fig. 3c clearly show that compared with GAC, the charge transfer resistance of GAC@Ni and GAC@Fe particle electrodes decreases and the ion diffusion rate increases, while the charge transfer resistance of GAC@Ni/Fe particle electrodes is the lowest. This increase in electrochemical performance can be attributed to the synergistic action of bimetals, which improves the conductivity of the composite material and reduces the internal resistance of the particle electrode. But from Fig. 3d we can also see that when the load metal ratio is 1:2 and 2:1, the charge transfer resistance increases, and only when the bimetal is 1:1 can a positive effect be achieved.

### Relationship between wetting state and electrochemical performance of particle electrode

**Figure 4:**
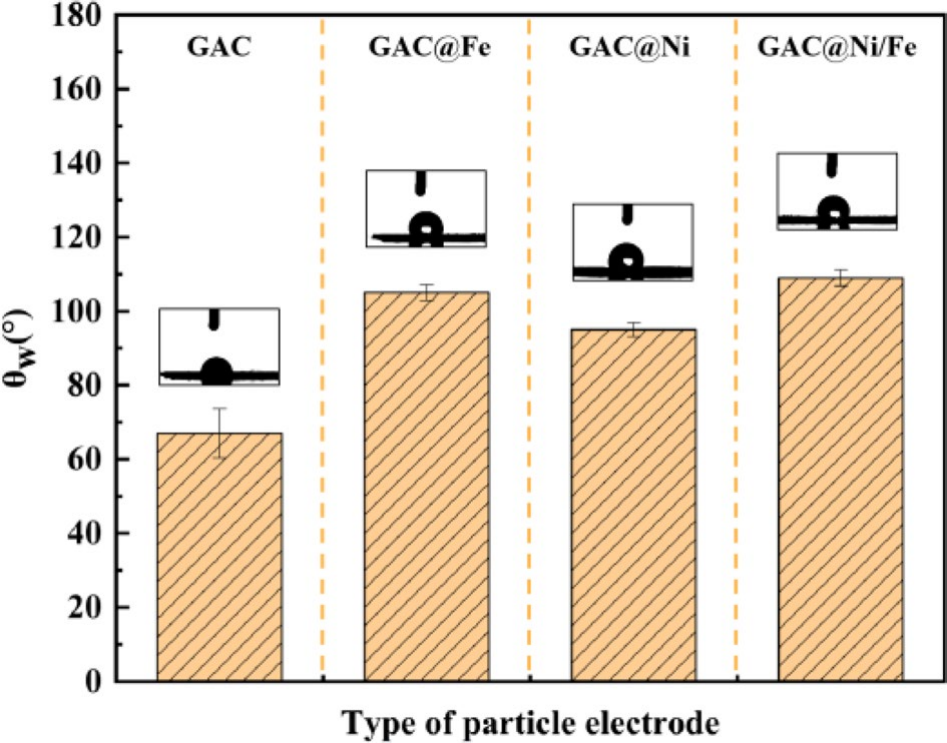
Wetting stability of particle electrodes loaded with different metals.

In recent years, it has been discovered that the wettability of the surface of the electrocatalyst can also be used to control the activity and selectivity of the chemical transformations involved. It is of particular concern in three-phase chemical systems where gaseous reactants, liquid electrolytes and solid catalysts (particle electrodes) are all in close contact^40,41^. As can be seen from Fig. 4, the θ_w_ = 67° ± 6° of activated carbon, which is not loaded with metal, is hydrophilic; After loading the metal, the θ_w_ is greater than 90° and becomes hydrophobic particle electrode in order of size θ_w(GAC@Ni)_ = 95° ± 1.9° < θ_w(GAC@Fe)_ = 105° ± 2.1° < θ_w(GAC@Ni/Fe)_ = 109° ± 0.9°。Compared with the test results of LSV curve and Tafel curve, the particle electrode exhibits higher electron transport capacity with the increase of hydrophobic contact Angle, which is consistent with the results of previous similar studies^42,43^. Compared with the test results of EIS, it is not difficult to find that the hydrophobic particle electrode shows a higher mass transfer rate, which also increases with the increase of θ_w_.

### Feasibility study on removal of toluene waste gas by three-dimensional electrode reaction device

**Figure 5:**
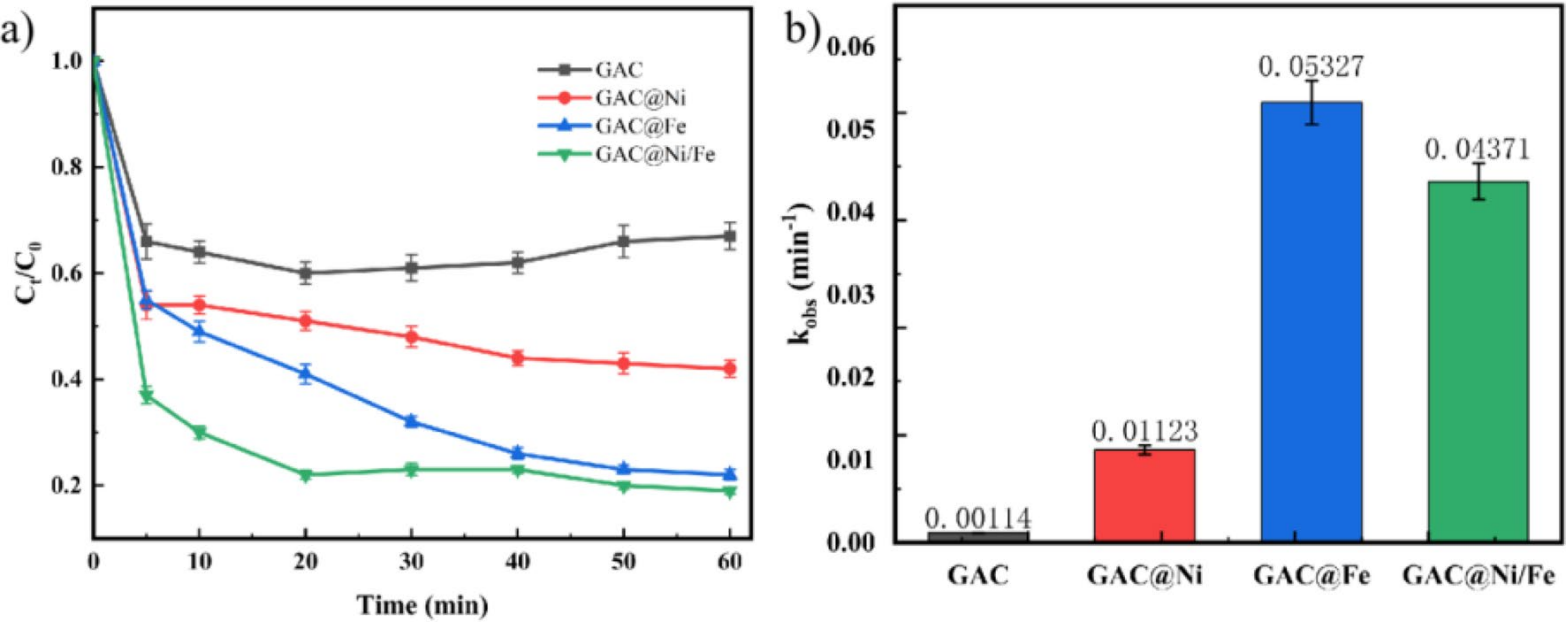
Effect of different metal-modified particle electrodes on toluene removal (**a**); degradation rate constants (**b**).

A three-dimensional electrode electrocatalytic oxidation device was constructed to test its effect on the removal of toluene waste gas. Adjust the air path to stabilize the flow rate before testing. The gas flow rate is 0.1 nL/min. In the three-dimensional electrode electrocatalytic oxidation device, the adsorption and removal of toluene in the device filled with 30 g different particle electrodes reached a stable state within 60 min. The main reason is that in the system, the adsorption of toluene by the particle electrode with adsorption effect reaches saturation. Because of its large specific surface area, GAC has the best adsorption and removal efficiency for gaseous toluene in the same time. The removal experiment began when toluene reached adsorption equilibrium, the anode and cathode were connected, and the power supply was switched on to form a closed loop. The concentration change of toluene in the three-dimensional electrode electrocatalytic system was shown in Fig. 5a. As can be seen from the figure, after the simulated waste gas containing toluene passes through the gas–liquid-solid three reaction zone, the GAC@Ni/Fe bimetallic catalytic particle electrode prepared in the filling has the best oxidation removal effect. After 1 h, the degradation efficiency gradually stabilized at 80%. Compared with the GAC particle electrode, the average degradation efficiency was increased by nearly 40%. Compared with the particle electrode loaded with Fe and Ni, the degradation efficiency was also increased by 9% and 24% respectively. These results show that the loading of metal can promote the production of more active substances in the three-dimensional electrode system to effectively degrade toluene waste gas, and improve the overall efficiency of the three-dimensional electrode system. Compared with the particle electrode loaded with single metal, bimetal iron and nickel have a synergistic effect on the degradation of pollutants^44^, which further strengthens the removal ability of gaseous pollutants. The study also carried out kinetic fitting of the degradation process using different particle electrodes, and the results were shown in Fig. 5b. It is not difficult to find from the figure that the degradation rate constant increases significantly after metal loading. The effect of metal iron loading alone is the best, and the lifting effect of nickel is low, so the rate constant of the reaction after both loading is also slightly lower than that of iron, but the degradation effect is better than that of single metal loading (The influence and interaction of key factors are detailed in the supporting materials). Compared with using the same electrochemical method to treat toluene waste gas in this study, the treatment capacity of this study has been greatly improved^45^. Although some other collaborative technologies involving electrochemistry have higher processing capacity, the process is relatively complicated and has higher requirements for reaction conditions and energy consumption. In summary, this study is more conducive to the expansion of application^46,47^.

In order to determine the use stability of the particle electrode, we also tested the reuse performance of the particle electrode. The experimental results are shown in Fig. S9. After 5 cycle experiments (10 h for each experiment), the degradation efficiency is still stable at more than 80%. When the degradation ability continues to decline, the electrocatalytic performance of the particle electrode can be recovered by repeated loading preparation process. In conclusion, the bimetallic GAC@Ni/Fe particle electrode prepared in this study is effective for the removal of toluene waste gas, and has the potential for reuse.

### Catalytic mechanism

Through the above study, it was found that the 3D electrode reactor filled with homemade GAC@Ni/Fe particle electrodes was more efficient in removing toluene. In order to further understand the mechanism of toluene exhaust gas degradation in the 3D electrode reactor and the role of filling the particle electrode in the reaction, the working principle of the 3D electrode reactor is analyzed in detail in this section. According to the empirical analysis of previous studies, the degradation of toluene in a three-dimensional electrode reactor may have the following three conditions. First, the electrode surface directly degrades the contaminants and oxidizes them. Secondly, the oxidized free radicals are generated by electrocatalytic oxidation to indirectly degrade the pollutants. Finally, the polarization of the GAC@Ni/Fe particle electrode in the 3D electrode reactor realizes the function of the microelectrolysis pool, while the adsorption and oxidation of pollutants, so as to improve the overall efficiency of the 3D electrode reactor.

The 3D electrode reactor used a self-made GAC@Ni/Fe particle electrode. In the process of electrocatalytic oxidation, metal ions on the surface of the particle electrode can activate hydrogen peroxide into hydroxyl radicals, thus degrading and removing gaseous pollutants that flow through the three-dimensional electrode reactor^48,49^. The reaction equation is as follows:

2 $$ {\text{O}}_{{2}} + {\text{ 2H}}^{ + } + {\text{ e}}^{ - } \to {\text{ H}}_{{2}} {\text{O}}_{{2}} $$

3 $$ {\text{Fe}}^{{{2} + }} + {\text{ H}}_{{2}} {\text{O}}_{{2}} + {\text{ H}}^{ + } \to {\text{ Fe}}^{{{3} + }} + \, \cdot{\text{OH}} + {\text{ H}}_{{2}} {\text{O}} $$

4 $$ {\text{Fe}}^{{{3} + }} + {\text{ e}}^{ - } \to {\text{ Fe}}^{{{2} + }} $$

5 $$ {\text{Ni}}^{{{3} + }} + {\text{ e}}^{ - } \to {\text{ Ni}}^{{{2} + }} $$

6 $$ {\text{Ni}}^{{{3} + }} + {\text{ Fe}}^{{{2} + }} \to {\text{ Ni}}^{{{2} + }} + {\text{Fe}}^{{{3} + }} $$

7 $$ \cdot{\text{OH}} + {\text{ Pollutants}} \to {\text{ CO}}_{{2}} + {\text{ H}}_{{2}} {\text{O}} $$

**Figure 6:**
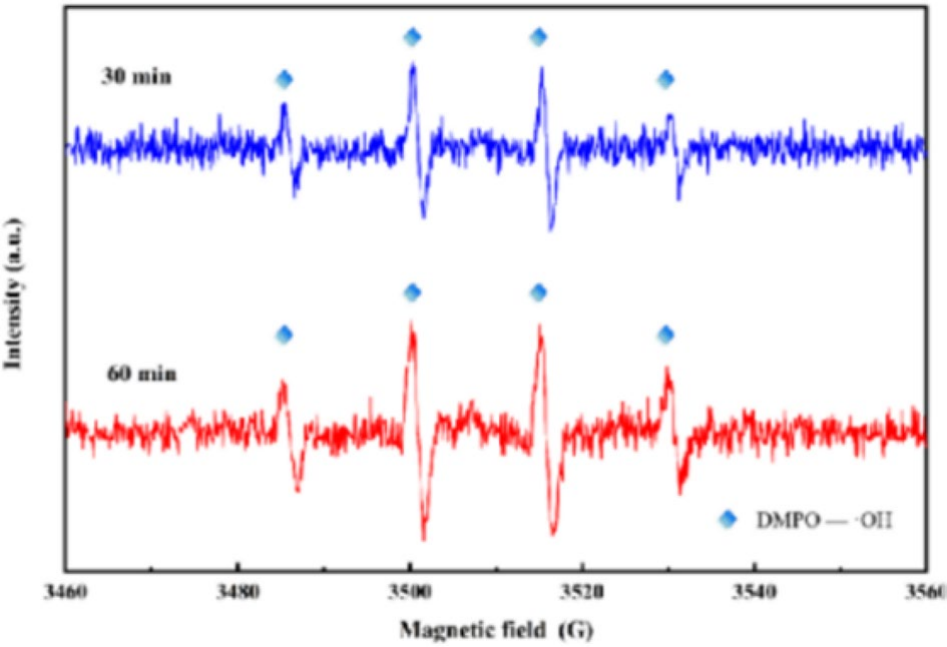
EPR spectra of ·OH.

To further determine the active species involved in toluene removal in a three-dimensional electrode reactor, EPR tests were performed. DMPO was used as the trapping agent to perform the test at 30 min and 60 min respectively. As shown in Fig. 6, we observed the typical spectrum of DMPO-·OH (peak intensity = 1:2:2:1), and the intensity of DMPO-·OH peak increased significantly with the increase of reaction time. ·OH radical is the main reactive substance for the removal of toluene by three-dimensional electrode in this study.

According to the test results and the reaction Eqs.(2)–(7), the degradation mechanism of toluene in the GAC@Ni/Fe composite electrode in the 3D electrode reactor is proposed. Toluene waste gas first enters the reactor from the bottom. When it flows through the anode, a small part of the waste gas is directly oxidized by the anode, and then enters the reaction zone of the particle electrode. Toluene waste gas is adsorbed by the GAC@Ni/Fe particle electrode, and the particle electrode is polarized. To achieve the effective removal of toluene waste gas, and finally generate non-toxic and harmless small molecules CO_2_ and H_2_O, the treated gas is discharged through the cathode. At the same time, we pass the discharged gas into the clarified lime water and observe that the clarified lime water becomes turbid. The production of CO_2_ is further proved. The cathode also produces oxidizing hydrogen peroxide, which indirectly promotes the formation of hydroxyl radicals.

### Degradation routes

Toluene molecule is relatively stable structure, the bond energy of C-H bond and C–C bond on benzene ring is 4.9 eV and 5.5 eV, respectively. The C–C bond energy of benzene ring and methyl group is 4.4 eV; The C-H bond on the methyl group has a bond energy of 3.7 eV^50^. First of all, according to the size of bond energy, we can preliminarily judge that high-energy electrons and free radical active species are more likely to attack C-H bonds with lower bond energy on methyl groups. By analogy, the possible reaction to toluene degradation is shown in Fig. S10^51^.

**Figure 7:**
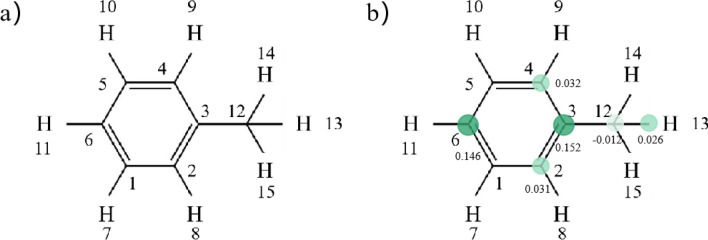
Molecular structure of toluene (**a**) and distribution of Fukui index (${f}^{0}$) on toluene (**b**).

**Figure 8:**
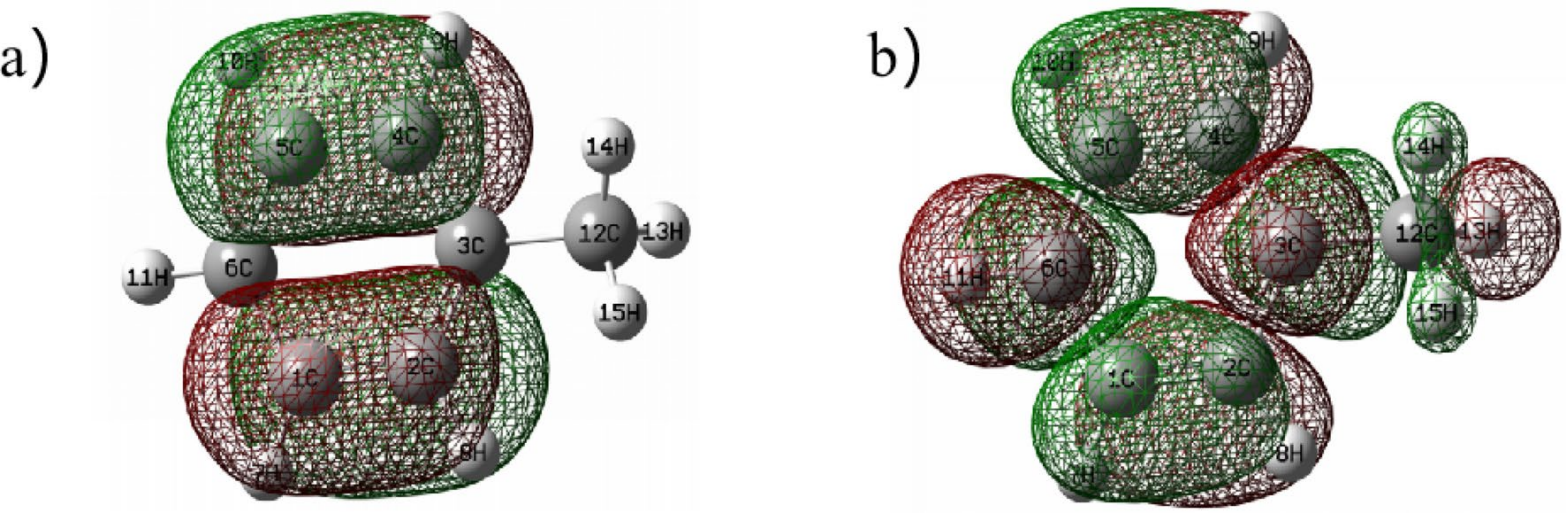
HOMO (**a**) and LUMO (**b**) of toluene molecules.

In order to further clarify the possible degradation pathway of toluene waste gas in the three-dimensional electrode reactor, solid phase extraction technology and GC–MS method were used to determine the electrolyte in the device, and the possible intermediate products were observed and analyzed. The test results are shown in Fig. S11. It can be seen from the products that a large amount of toluene is dissolved in the electrolyte, and the degradation products of toluene can be divided into two classes, one is p-Xylene, o-Cymene, p-Cymene, Benzaldehyde, 2-Amino-4-methylbenzoic acid and other compounds containing benzene rings. The other is the small molecule product formed after the benzene ring is broken or recombined compounds, etc. At the same time, the toluene molecule was calculated theoretically. The structure of toluene molecule was optimized with Gaussian 09 software based on 6-31G group under b3lyp theory ^52^. The optimized structure is shown in Fig. S12. The NBO charge is then calculated, and the charges of natural population analysis (NPA) on SMT in different electronic states are shown in Table 1. Thus, the size of the compressed Fukui function for each atom in the molecule is further calculated, and the distribution of Fukui index (${f}^{0}$) on SMT is shown in Fig. 7 Combined with the molecular electrostatic energy Fig. S13, the interaction forces between molecules can be illustrated. Gaussview 5.0 was used to draw a 3D map of MEPs of toluene molecules after optimization. The red area represents the electronegativity, electrophilic correlation, and the blue area represents the electronegativity, nucleophilic correlation, and the higher the degree of color, the greater the trend. As shown in Fig. 8, E_HOMO_ = − 0.0.47036 a.u., E_LUMO_ = − 0.23744 a.u. The low HOMO energy of toluene indicates that its HOMO orbital electron has a weak ability to give electrons, while the high LUMO orbital energy indicates that its LUMO orbital electron has a strong ability to accept electrons^53^. It can be inferred that toluene underwent a process of ring-opening by oxidation of ·OH and further formation of short-chain compounds, and finally into CO_2_ and H_2_O^54^ during the degradation process.

**Table 1:** Natural population Analysis (NPA) of charge distribution on toluene molecules and calculated Fukui index (${f}^{0}$) levels.

No	Atom	Charge(0) (e/Å^3^)	Charge(+ 1) (e/Å^3^)	Charge( − 1) (e/Å^3^)	${f}^{0}$
1	C	− 0.237	− 0.127	− 0.096	0.015
2	C	− 0.247	− 0.115	− 0.053	0.031
3	C	− 0.036	− 0.022	0.282	0.152
4	C	− 0.247	− 0.115	− 0.050	0.032
5	C	− 0.237	− 0.127	− 0.099	0.014
6	C	− 0.258	− 0.095	0.198	0.146
7	H	0.246	0.117	0.147	0.015
8	H	0.242	0.119	0.143	0.012
9	H	0.242	0.119	0.143	0.012
10	H	0.246	0.117	0.147	0.015
11	H	0.246	0.112	0.138	0.013
12	C	− 0.696	− 0.336	− 0.359	− 0.012
13	H	0.250	0.118	0.169	0.026
14	H	0.244	0.118	0.145	0.014
15	H	0.243	0.118	0.145	0.014

## Conclusion

In this study, the GAC@Ni/Fe bimetal particle electrode was prepared by surface control of nickel and iron bimetal through defect engineering, and the toluene waste gas was effectively removed by three-dimensional electrode reaction device. The electrochemical active surface area (ECSA) and mass transfer rate (K) of the conventional GAC particle electrode were effectively increased by the analysis of the cyclic voltammetry curve (CV). The linear voltammetry curve (LSV) shows that GAC@Ni/Fe particle electrode has higher electrocatalytic activity, and the Tafel curve shows that it can achieve faster electron transport kinetic rate, and the best effect is when the bimetal ratio is 1:1. At the same time, it is also verified that with the increase of hydrophobic contact Angle, the particle electrode shows a higher electrode electron transport capacity. In this study, the central composite design (CCD) was used to optimize the operation of tank voltage, particle electrode dosage and electrolyte concentration. In this study, the optimal removal efficiency of p-toluene waste gas could be achieved when the tank voltage was 15 V, the particle electrode dosage was 50 g and the electrolyte concentration was 1 mol/L. At the same time, the active species of toluene effectively removed by hydroxyl radical in the three-dimensional electrode reaction device were clarified through the free radical capture experiment, and the process of toluene being oxidized by ·OH to open the ring and further generate short chain compounds, and finally oxidized to water and carbon dioxide was analyzed. This study provides a new possibility for the application of three-dimensional electrode reaction device to remove VOCs in the future, and effectively supplements the theoretical research.

### Supplementary Information

Supplementary Information.

## Data Availability

The datasets generated and/or analysed during the current study are available from the corresponding author on reasonable request.
