# Supplementary material for: A new attempt to remove toluene using nickel–iron bimetallic particle electrode reactor
Source: Sci Rep. 2024 May 2;14:10056. doi: 10.1038/s41598-024-60956-0 (PMC11065997; doi:10.1038/s41598-024-60956-0)
Supplement: Supplementary file 1 — Supplementary Information. [file 41598_2024_60956_MOESM1_ESM.docx]

**Supplementary material**

The GAC@Ni/Fe particle electrode was prepared by liquid phase reduction by defect control. Defect engineering is an effective method to regulate the surface properties of materials and improve the electrocatalytic activity of reactions, which has important potential to study the interface effect of catalysts ^1^. Controlled structural defects in electrode materials can not only effectively promote ion diffusion and charge transfer, but also provide more storage sites / adsorption sites / active sites for metal ions or intermediates, which helps to maintain the structural flexibility and stability of the material. Defect engineering has become an important research direction of carbon-based electrocatalysts recently. The carbon defect site can be directly used as the active site due to the change in the surface charge state, the adsorption free energy of the intermediate and the decrease of the band gap. In addition, the synergistic effect between intrinsic defects and heteroatom doping can further optimize the electronic structure and adsorption / desorption behavior, making carbon-based catalysts comparable to commercial precious metal catalysts in electrocatalysis ^2^.


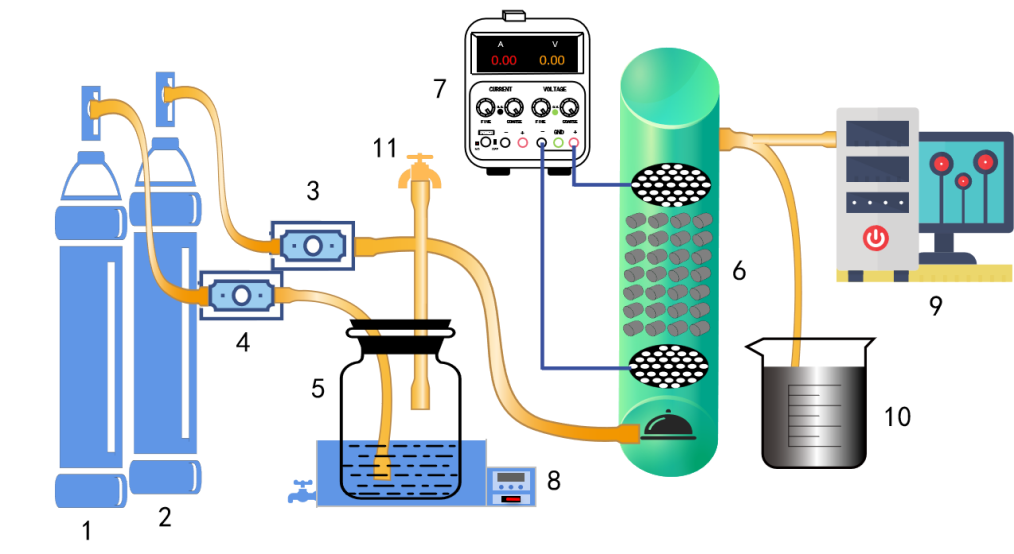


**Fig. S1.** Schematic diagram of the experimental setup (1: oxygen cylinder; 2: nitrogen cylinder; 3, 4: flow meter; 5: Toluene bottle; 6: three-dimensional electrode reaction device; 7: DC power supply; 8: Water bath; 9: Gas chromatograph; 10: exhaust gas absorption bottle; 11: Valve)

### Analysis of mass transfer rate and electrochemical active surface area

In this study, the scan rates were controlled as follows: 0.2 V/s, 0.1 V/s, 0.05 V/s and 0.01 V/s, respectively. The cyclic voltammetry curves (CV) of GAC, GAC@Ni, GAC@Fe and GAC@Ni/Fe particle electrodes and the cyclic voltammetry curves (CV) of GAC@Ni/Fe particle electrodes loaded with different proportions of Ni and Fe metal were tested respectively. According to the research of Ni et al. ^3^, the cyclic voltammetry curve can be used to measure the mass transfer rate (k) and electrochemical active surface area (ECSA) of the electrode. Good linear relationship between the peak oxidation current (I_P_) and the square root (V^1/2^) of the scan rate: I_P_ = kV^1/2^. Thus, the peak oxidation current (I_P_) and the square root (V^1/2^) of the scan rate are fitted linearly, the results of which are shown in Fig. 2 and Table 1.

Through experiments, we found that the mass transfer rate of GAC particle electrode k_GAC_=0.3221, when loaded with single metal, the mass transfer rates of GAC@Ni and GAC@Fe are increased to k_GAC@Ni_=0.3403 and k_GAC@Fe_=0.3869, respectively. When loaded with bimetal simultaneously, the mass transfer rate of GAC@Ni/Fe particle electrode is 1.29 times that of pure GAC particle electrode. k_GAC@Ni/Fe_=0.4166. This shows that different metal loads have a good effect on improving the mass transfer rate of conventional GAC particle electrodes. At the same time, it can be concluded that the electrochemical active surface area (ECSA) of the GAC@Ni, GAC@Fe and GAC@Ni/Fe particle electrodes are 1.06,1.2 and 1.29 times that of the GAC particle electrode, respectively. Meanwhile, it is also said that the load of metal effectively improves the electrochemical active surface area of the traditional GAC particle electrode. However, according to the apparent data, the load of single metal nickel does not improve the electrochemical active surface area (ECSA) significantly, which is lower than the simultaneous load of single metal iron and bimetal metal. At the same time, we investigated the improvement of the electrochemical active surface area of two kinds of Ni and Fe metal load ratios for particle electrodes. The experimental results are shown in Fig. 3 The electrochemical properties of the particle electrode are best improved when the bimetallic load ratio is 1:1.

**Table S1** Linear fitting equation of I_P_ and V^1/2^ and mass transfer rate

| **Particle electrode** | **I_P_ and V^1/2^ linear fitting equation** | **R^2^** | **Mass transfer rate（k）** |
| --- | --- | --- | --- |
| GAC | y=0.3221x+0.2504 | 0.9041 | 0.3221 |
| GAC@Ni | y=0.3403x+0.3662 | 0.9587 | 0.3403 |
| GAC@Fe | y=0.3869x+0.3191 | 0.9581 | 0.3869 |
| GAC@Ni/Fe（1：1） | y=0.4166x+0.3708 | 0.9694 | 0.4166 |
| GAC@Ni/Fe（1：2） | y=0.3407x+0.3771 | 0.8987 | 0.3407 |
| GAC@Ni/Fe（2：1） | y=0.2141x+0.2101 | 0.9665 | 0.2141 |


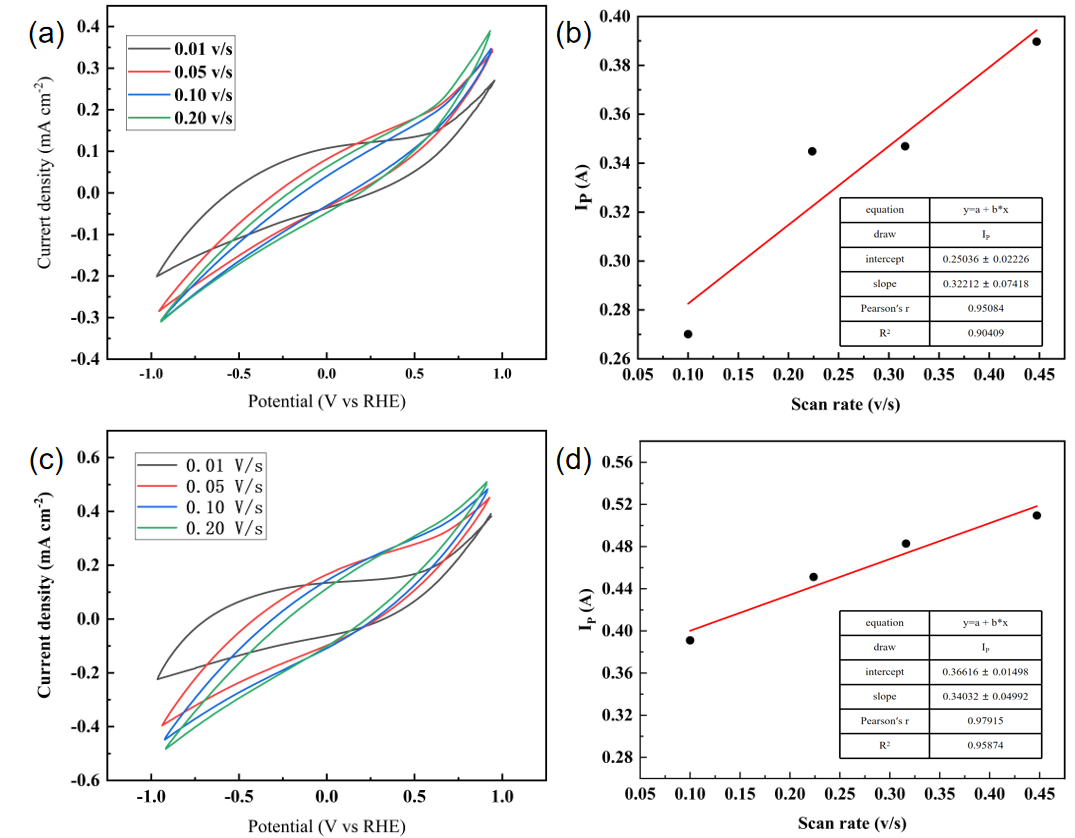

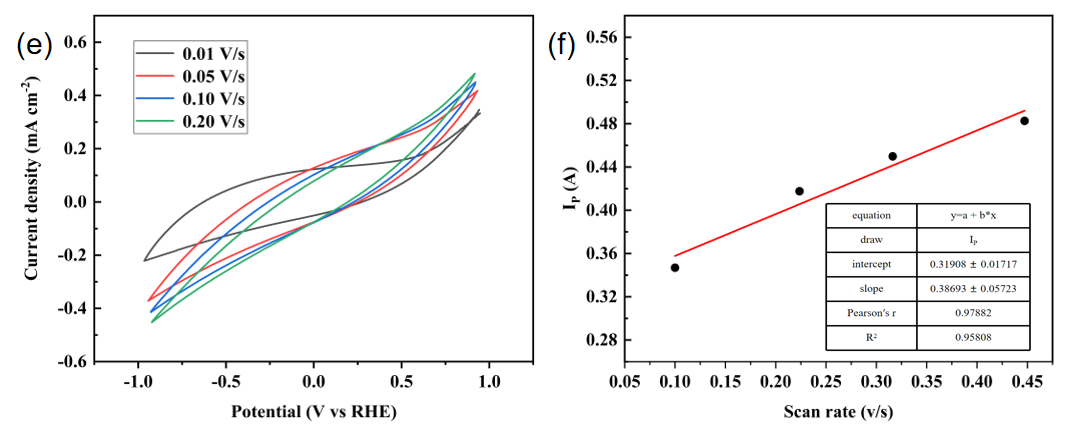


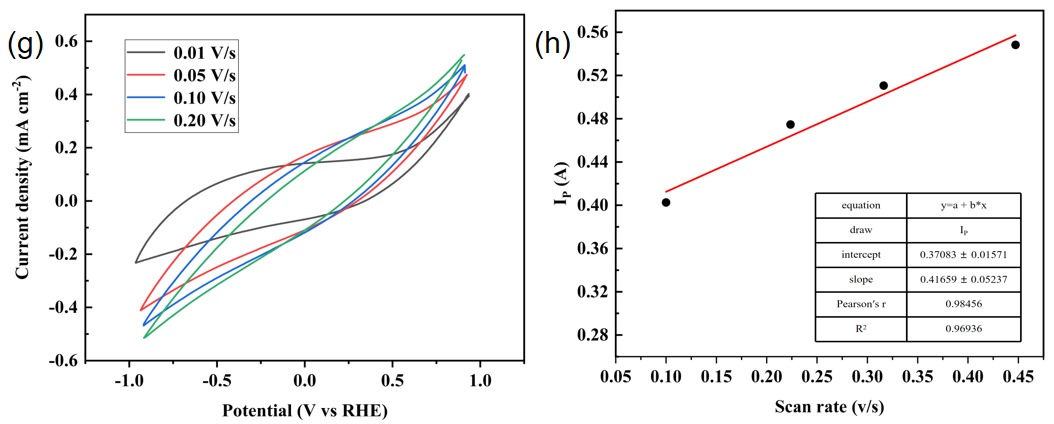


**Fig. S2.** Cyclic voltammetry (CV) curve (a) and I_P_ and V^1/2^ linear fitting curve (b) of GAC particle electrode; GAC@Ni cyclic voltammetry (CV) curve (c) and linear fitting curve (d) of I_P_ and V^1/2^ for particle electrodes; GAC@Fe cyclic voltammetry (CV) curve (e) and linear fitting curve (f) of I_P_ and V^1/2^ for particle electrodes; Cyclic voltammetry (CV) curve (g) and linear fitting curve (h) of I_P_ and V^1/2^ for GAC@Ni/Fe particle electrode

We explored the improvement of the electrochemical activity surface area of the particle electrode by Ni and Fe metals, and the experimental results are shown in Figure 1. As we can see from the following figure, k_GAC@Ni/Fe(1:1)_=0.4167, k_GAC@Ni/Fe(2:1)_=0.3407, k_GAC@Ni/Fe(1:2)_=0.2141, the change of bimetallic load ratio also has a significant effect on the mass transfer rate of the particle electrode. It is obvious that the k value is the maximum when the bimetal load ratio is 1:1. Its electrochemical active surface area (ECSA) is also 1.22 and 1.95 times that of the other two. Therefore, when the load ratio of 1:1 improves the electrochemical performance of the particle electrode best, and the synergistic efficiency of the bimetals is the greatest.


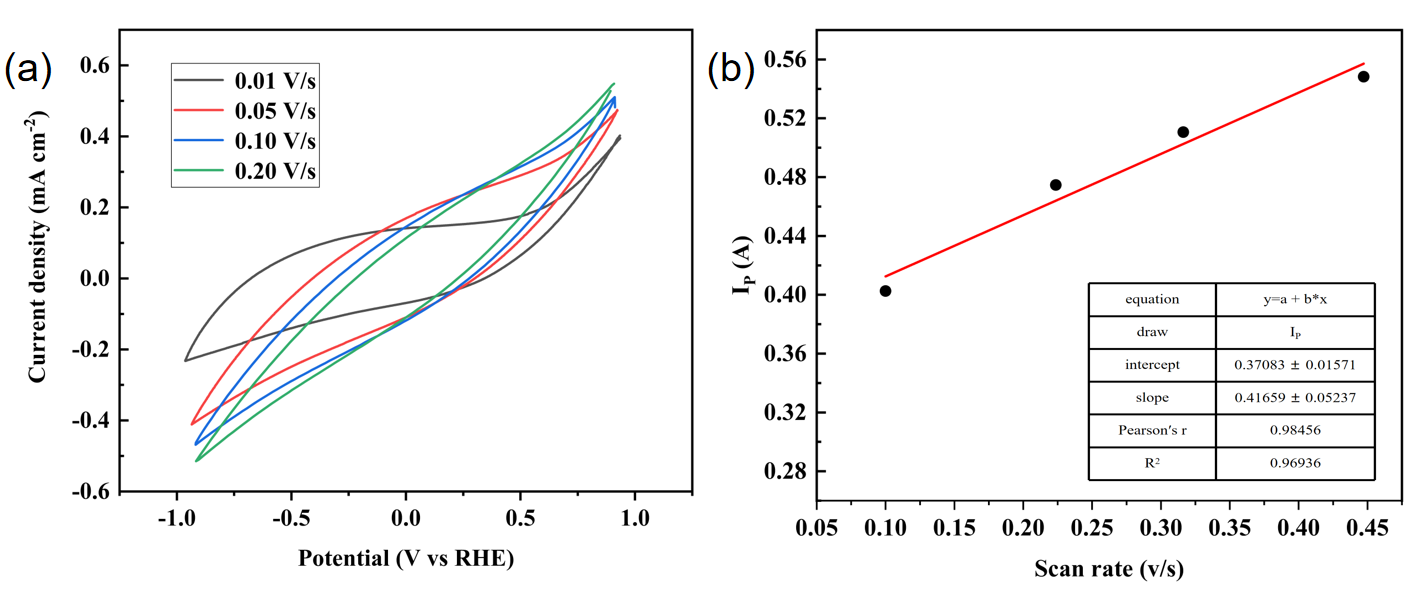


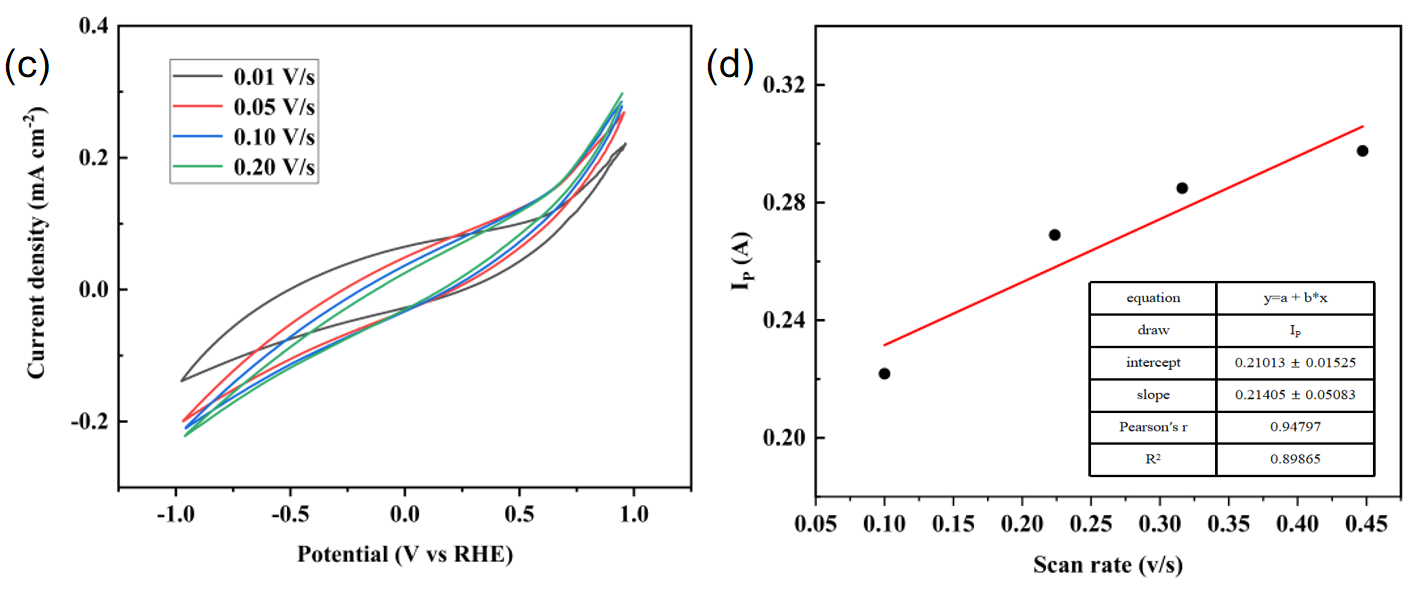


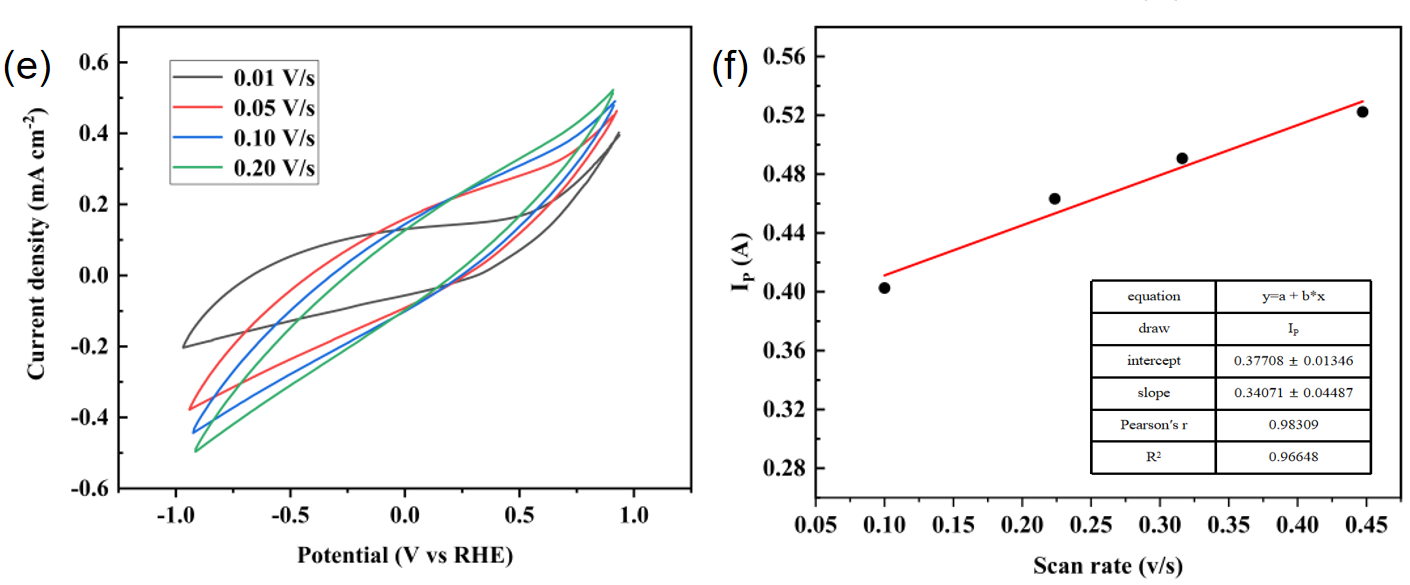


**Fig. S3.** Cyclic voltammetry (CV) curve (a) and linear fitting curve (b) of I_P_ and V^1/2^ for GAC@Ni/Fe(1:1) particle electrode; Cyclic voltammetry (CV) curve (c) and linear fitting curve (d) of I_P_ and V^1/2^ for particle electrode GAC@Ni/Fe(2:1); GAC@Ni/Fe(1:2) Cyclic voltammetry (CV) curve (e) and linear fitting curve (f) for I_P_ and V^1/2^ particle electrodes


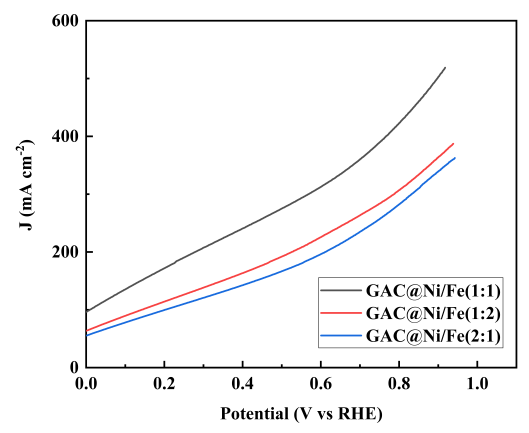


**Fig. S4.** Linear voltammetry (LSV) curves of particle electrodes with different load metal proportions

### Key factor influences

For the practical application of three-dimensional electrode reactor in the field of VOCs removal, we need to preliminarily understand the influence trend of basic operating conditions on reactor performance. The removal efficiency of the reactor can be significantly affected by the highly controllable basic operating conditions such as the variation of tank voltage, the amount of particle electrode, gas residence time and electrolyte concentration. Therefore, the above conditions are analyzed in this study.

**Effect of the slot voltage.** When other conditions of the reaction system remain unchanged, the tank voltage will affect the current density of the system, and thus directly affect the removal efficiency of toluene. In this study, the regulating tank voltage increased from 3 V to 15 V, and the results are shown in Fig. 5a. From the figure, we can find that when the voltage varies between 3 V-9 V, the removal efficiency is almost the same within the error range. The main reason is that the anode and cathode of the reactor are fixed, relatively far away, the electrolyte concentration and the particle electrode amount are fixed, and the current density and the effect on the particle electrode polarization are relatively weak at relatively low tank voltage ^4^, so the direct reaction is only about 24 % in terms of removal rate. With the continuous increase of voltage, the removal capacity of the reactor shows more excellent performance. When the voltage is 12 v, the removal capacity is 1.62 times as before, and when the voltage is continued to increase to 15 V, the removal capacity is 3.31 times as before, and the average removal efficiency is 82.5 %. After the current density increases, more particle electrodes repolarization, more micro-electrolytic cells are generated, and the REDOX effect on the surface of the particle electrode is enhanced. It can also significantly accelerate the production of ·OH ^5^.

**Effect of particle electrode injection.** As shown in Fig. 5b. In this study, as the amount of particle electrode in the three-dimensional electrode reactor increased from 10 g to 50 g, the average removal efficiency of the reactor increased from 40 % to 84 % after 60 min. The increase of the amount of particle electrode directly leads to the increase of the surface area of particle electrode and the active site in the reaction process, and more free radicals are generated for the degradation of pollutants through the electrocatalytic reaction, and the reaction efficiency continues to increase. However, in previous studies and analyses, it was found that in three-dimensional electrode reactors, with the continuous increase in the amount of particle electrodes, the particle electrodes tended to accumulate and lead to the generation of short circuit current, and the reaction efficiency basically showed a trend of first increasing and then decreasing ^6^. Three currents in the system, namely reaction current, short circuit current and bypass current, the reaction current works. Excessive particle addition will also take up more reactive space, reduce the efficiency of organic pollutant mass transfer, while increasing short circuit current and reducing current efficiency ^7^. In this study, the packed bed structure is adopted, and the particle electrode is effectively isolated from the anode and cathode, as well as each other, and the short circuit current is effectively reduced. Therefore, with the increase of particle electrode in the reaction device, the reaction efficiency continues to increase.

**Effect of the gas flow rate.** In the continuous flow experiment, the gas flow rate can affect the contact time of pollutants in the reaction device and the mass transfer effect, which is reflected in the removal ability of pollutants. The inlet flow rate was changed, and the experimental results were shown in Fig. 5c. It can be seen from the figure that when the intake rate is low, the removal efficiency of toluene is higher. With the increase of the rate, the removal capacity of toluene decreases linearly, the volume of the reaction device is fixed, and the residence time decreases with the increase of the flow rate. When it rises to 0.4 nL/min, the removal capacity is only 2 %. It can be seen that the residence time of pollutants in the device plays a crucial role in the removal capacity of the reaction device, which also provides one of the key contents for the future application of electrochemistry in the field of VOCs removal and the development and design of the reactor.

**Effect of the electrolyte concentration.** This is shown in Fig. 5d. After the reaction time of 60 min, the removal efficiency of toluene gradually stabilized under different electrolyte concentrations. When the concentration of sodium sulfate increased from 0.1 mol L^−1^ to 0.2 mol L^−1^, the removal efficiency of the reactor increased from 62 % to 82 %, an increase of nearly 20 %. As the concentration of sodium sulfate continued to increase to 0.5 mol L^−1^, the removal efficiency continued to decrease. One of the main factors leading to this result is that the bypass current of the reaction system increases and the reaction current decreases due to high electrolyte concentration and high conductivity ^8^. In addition, too high concentration will also cause waste of electrolyte and increase the difficulty of subsequent pollutant treatment ^9^. Secondly, the increase of electrolyte concentration has limited effect on the gain of current efficiency. When there are too many cationic ions in the solution, they will gather on the anode and cathode surface and the particle electrode surface, hindering the electron gain and loss process on the electrode surface, greatly reducing the number of effective active sites on the electrode surface and hindering the generation of ·OH. And the increase of electrolyte concentration leads to the increase of conductivity, and the current also increases, resulting in side reactions. For example, the hydrogen evolution reaction produces a large number of bubbles, which affects the electrocatalytic degradation and aggravates the power consumption. Therefore, in the experimental design of this study, the optimal concentration of electrolyte is 0.2 mol L^−1^. In the design and use of the device, it does not mean that the higher the electrolyte concentration is more conducive to the reaction, and controlling and adjusting the concentration parameters of the electrolyte is also one of the important factors to improve the efficiency of the device.


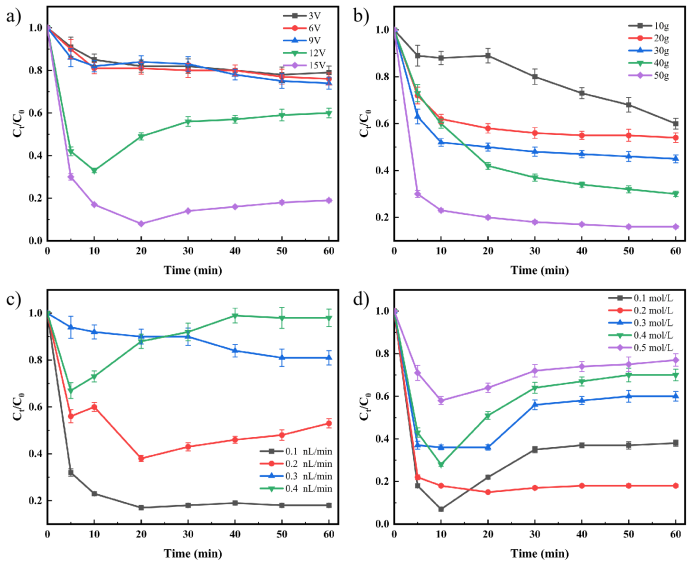


**Fig. S5.** Influence of different operating conditions on toluene removal Slot voltage (a); Particle electrode dosage (b); Gas flow rate (c); Electrolyte concentration (d)

### Interaction mechanism and optimization of influencing factors

In this study, the relationship between independent parameters and dependent variables was analyzed by central composite design (CCD) and operational parameters were optimized. A quadratic model of the response surface was generated by ANOVA to explain the model fit, accuracy, and importance, as well as the effect of interaction results and individual parameters on response values ^10,11^. The experimental design matrix and the results are shown in Table 2. Through the fitting analysis of the experimental results, the empirical relationship between the response and the independent variables is shown in Eqs. (1) :

Y=28.52-0.40A-0.36B-18.97C+0.037AB-3.04AC+0.39BC+0.18A^2^+0.005B²+31.57C² (1)

Where Y is toluene removal ratio (%), A is voltage (V), B is particle electrode dosage, and C is electrolyte concentration.

In order to further verify the accuracy and adequacy of the toluene degradation model, analysis of variance (ANOVA) was conducted, as shown in Table S3.

**Interactions of the operating conditions.** Fig. 6a and 6b show the contour diagram and response surface diagram of toluene removal at different voltages and particle electrodes when the electrolyte concentration is 0.1 mol/L. When the amount of particle electrode is the same, the removal efficiency of toluene increases with the increase of voltage. When the particle electrode content is less, the increase of voltage has no obvious effect on the degradation efficiency, but the change is obvious when the particle electrode content is more. Therefore, the optimal amount of voltage and particle electrode can be inferred from the response surface and contour plot based on the economic cost and efficiency of the practical application. Fig. 6c and 6d show the contours and response surfaces of toluene removal at different voltages and electrolyte concentrations when the particle electrode is 30 g. When the electrolyte concentration is low, the range of voltage change has great influence on the degradation efficiency. The higher the voltage, the higher the degradation efficiency and the more obvious the change. When the electrolyte concentration is high and the voltage is high, side reactions that hinder the degradation efficiency will also occur in the system, so the interaction analysis of the two can effectively adjust the degradation efficiency of the system. Fig. 6e and 6f show the contours and response surfaces of the particle electrode dosage and electrolyte concentration at a slot voltage of 15 V. With the increase of electrolyte concentration, the degradation efficiency of toluene increased first and then decreased. The more particle electrodes were added, the more obvious the degradation efficiency was. Combined with ANOVA, the interaction between the two was not very significant.

**Fig. S6.** Degradation efficiency of toluene waste gas predicted by CCD (%) Contour diagram (a) and response surface diagram (b) of tank voltage and particle electrode dosage; Contours of cell voltage and electrolyte concentration (c) and response surface (d); Contour diagram (e) and response surface diagram (f) of particle electrode dosage and electrolyte concentration

**Process condition optimization and verification.** The main purpose of this study is to improve the degradation ability of the three-dimensional electrode reaction device for VOCs, so based on the test results of this study, we carried out fitting optimization of the RSM model. The optimum degradation conditions for the maximum degradation variables were as follows: cell voltage =15 V, particle electrode dosage =50 g, electrolyte concentration =1 mol/L. The degradation efficiency was 81.72 % (Desirability=0.769). In order to further determine the structure of the simulation, we conducted three experiments under the condition of optimal solution, and the experimental results were very close to the predicted results of the fitted model (the error is less than 0.5%), which verified the effectiveness of the expected model (Fig. 8).

**Table S2 Experimental design matrix and experimental results**

| Std | Run | Factor 1  A：Slot voltage  V | Factor 2  B：Particle electrode dosage  g | Factor 3  C：Electrolyte concentration  mol/L | Response 1  Toluene removal rate  % |
| --- | --- | --- | --- | --- | --- |
| 19 | 1 | 9 | 30 | 0.3 | 42.9 |
| 18 | 2 | 9 | 30 | 0.3 | 35.23 |
| 3 | 3 | 3 | 50 | 0.1 | 30.3 |
| 5 | 4 | 3 | 10 | 0.5 | 24.68 |
| 13 | 5 | 9 | 30 | -0.0363586 | 42.37 |
| 8 | 6 | 15 | 50 | 0.5 | 72.74 |
| 11 | 7 | 9 | -3.63586 | 0.3 | 26.4 |
| 16 | 8 | 9 | 30 | 0.3 | 33.56 |
| 15 | 9 | 9 | 30 | 0.3 | 32.22 |
| 4 | 10 | 15 | 50 | 0.1 | 82.56 |
| 7 | 11 | 3 | 50 | 0.5 | 33.07 |
| 10 | 12 | 19.0908 | 30 | 0.3 | 84.3 |
| 1 | 13 | 3 | 10 | 0.1 | 26.1 |
| 12 | 14 | 9 | 63.6359 | 0.3 | 52.35 |
| 2 | 15 | 15 | 10 | 0.1 | 62.38 |
| 14 | 16 | 9 | 30 | 0.636359 | 32.49 |
| 9 | 17 | -1.09076 | 30 | 0.3 | 20.72 |
| 17 | 18 | 9 | 30 | 0.3 | 35.89 |
| 6 | 19 | 15 | 10 | 0.5 | 44.35 |

**Table S3 Analysis of Variance of the response surface quadratic model (ANOVA)**

| **Source** | **Sum of Squares** | **df** | **Mean Square** | **F-value** | **P-value** |  |
| --- | --- | --- | --- | --- | --- | --- |
| **Model** | 6589.60 | 9 | 732.18 | 59.75 | < 0.0001 | significant |
| A | 4754.19 | 1 | 4754.19 | 387.98 | < 0.0001 |  |
| B | 804.25 | 1 | 804.25 | 65.63 | < 0.0001 |  |
| C | 136.12 | 1 | 136.12 | 11.11 | 0.0088 |  |
| AB | 161.82 | 1 | 161.82 | 13.21 | 0.0055 |  |
| AC | 106.58 | 1 | 106.58 | 8.70 | 0.0162 |  |
| BC | 19.22 | 1 | 19.22 | 1.57 | 0.2420 |  |
| A² | 593.56 | 1 | 593.56 | 48.44 | < 0.0001 |  |
| B² | 51.92 | 1 | 51.92 | 4.24 | 0.0697 |  |
| C² | 21.76 | 1 | 21.76 | 1.78 | 0.2154 |  |
| **Residual** | 110.28 | 9 | 12.25 |  |  |  |
| Lack of Fit | 41.83 | 5 | 8.37 | 0.4889 | 0.7739 | not significant |
| Pure Error | 68.45 | 4 | 17.11 |  |  |  |
| **Cor Total** | 6699.88 | 18 |  |  |  |  |

Factor coding is Coded. Sum of squares is Type III - Partial. R^2^=0.9835；R^2^_daj_=0.9671

The Model F-value of 59.75 implies the model is significant. There is only a 0.01 % chance that an F-value this large could occur due to noise. P-values less than 0.0500 indicate model terms are significant. In this case A, B, C, AB, AC, A² are significant model terms. Values greater than 0.1000 indicate the model terms are not significant. The Lack of Fit F-value of 0.49 implies the Lack of Fit is not significant relative to the pure error. There is a 77.39 % chance that a Lack of Fit F-value this large could occur due to noise. The Predicted R² of 0.9364 is in reasonable agreement with the Adjusted R² of 0.9671; i.e. the difference is less than 0.2. Adeq Precision measures the signal to noise ratio. A ratio greater than 4 is desirable. This study ratio of 24.853 indicates an adequate signal. This model can be used to navigate the design space.

(a)
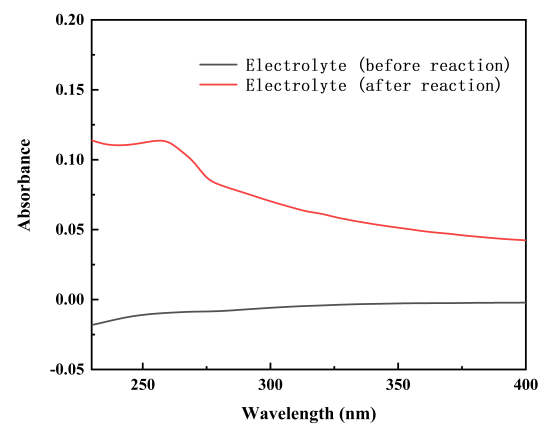
(b)
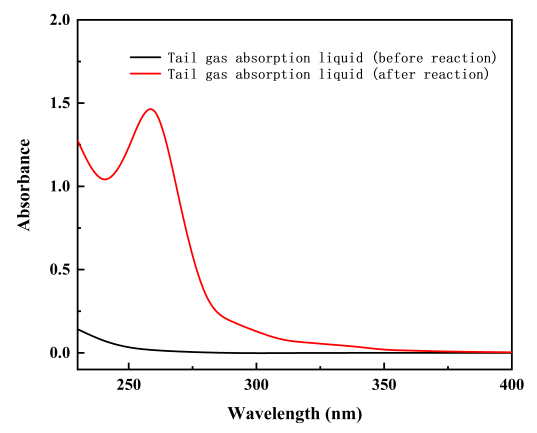


**Fig. S7.** Ultraviolet spectrophotometer before and after the reaction of electrolyte (a) with tail gas absorber (b)


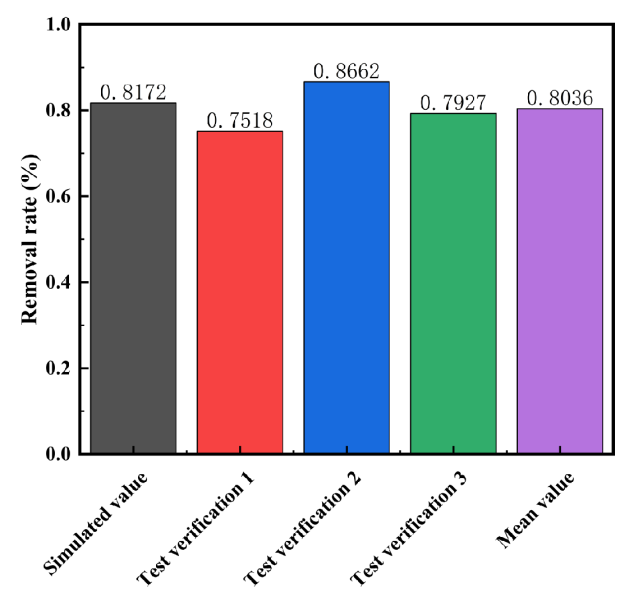


**Fig. S8.** Comparison of simulated values and test validation values

### Particle electrodes reusability and stability

In order to evaluate the long-term stability of GAC@Ni/Fe particle electrode, the average removal efficiency of toluene exhaust gas from the particle electrode was measured every 10 hours at a 2-hour interval during each cycle. The experimental results are shown in Fig. 9. From the figure, we can see that the average removal efficiency in the first 10 hours is 81.0%, and in the following 4 cycles, the average removal efficiency is 84.0%, 81.0%, 82.0% and 82.0%, respectively. At the beginning of the sixth cycle, the average removal efficiency began to decrease to 77.0% and at the beginning of the seventh cycle to 71.0%. Its removal efficiency is still significantly better than that of traditional GAC particle electrode in three-dimensional electrode reactor. In conclusion, the bimetallic GAC@Ni/Fe particle electrode prepared in this study is effective for the removal of toluene waste gas and can be reused.


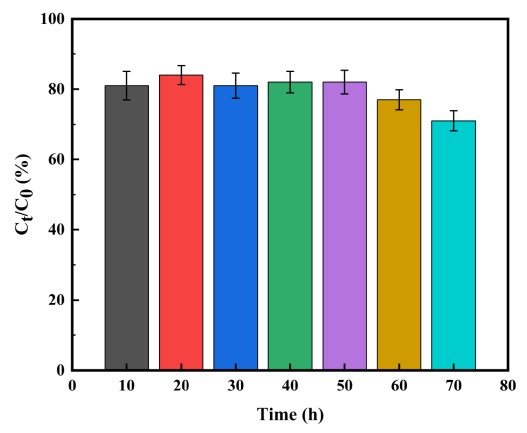


**Fig. S9.** Analysis of the recycling effect of GAC @ Ni/Fe particle electrode

**Fig. S10.** Possible reactions of toluene degradation

**Table S4** Intermediate products of degradation of toluene in electrolyte and tail gas absorption solution

| **Run** | **Intermediate product** | **Structural formula** |
| --- | --- | --- |
| 1 | Benzaldehyde | 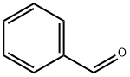 |
| 2 | p-Xylene | 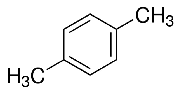 |
| 3 | o-Cymene | 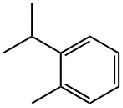 |
| 4 | p-Cymene | 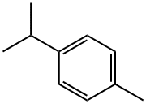 |
| 5 | 2-Amino-4-methylbenzoic acid | 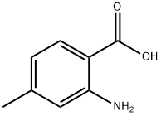 |


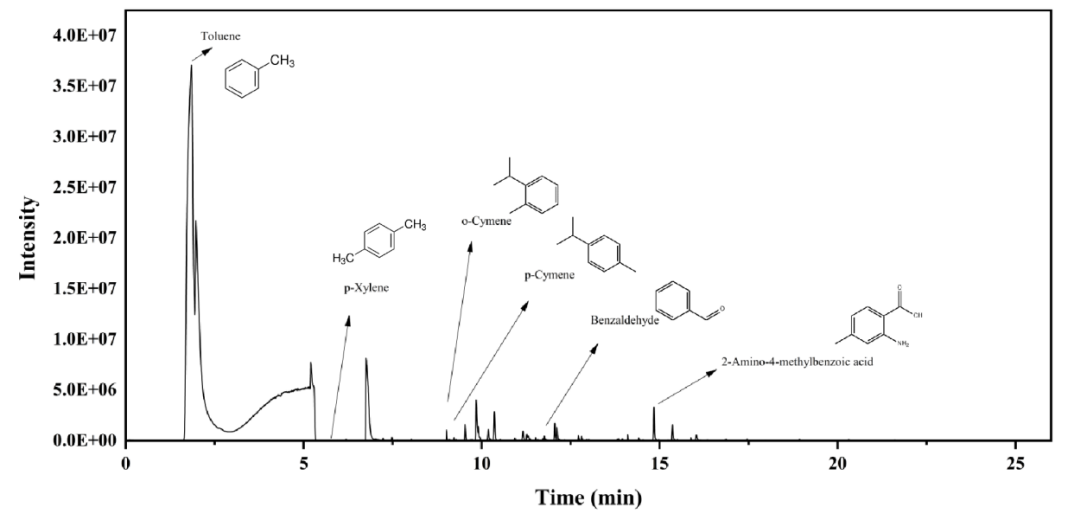


**Fig. S11.** Analysis of toluene degradation products by GC-MS


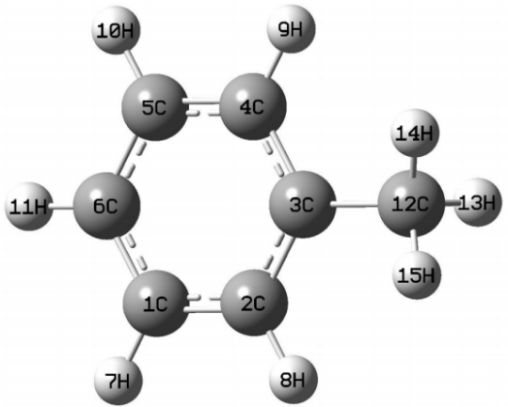


**Fig. S12.** The optimized configuration diagram of toluene molecule


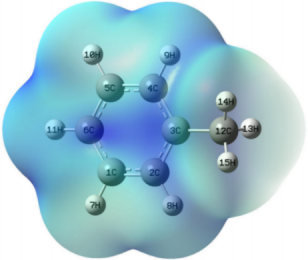

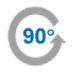

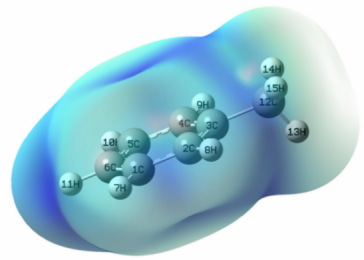


**Fig. S13.** 3D diagram of MEPs of toluene molecule

# Reference

1. Wang, S., Li, H., Chen, C., Yan, D., & Zou, Y. . Interfacial effects in supported catalysts for electrocatalysis. Journal of Materials Chemistry A. 7(7), (2019).
2. Zhu, J. , & Mu, S. Defect engineering in the carbon‐based electrocatalysts: insight into the intrinsic carbon defects. Advanced Functional Materials (2020).
3. Ni Jin. Study on the Effect of Loaded y-MnO_2_ Particles on the Degradation of Rhodamine B Wastewater by Three-dimensional Electrode Reactor [D], University of Science and Technology Beijing. (2021).
4. Pang, T. , Wang, Y. , Yang, H. , Wang, T. , & Cai, W.Dynamic model of organic pollutant degradation in three dimensional packed bed electrode reactor. Chemosphere, 206(SEP.), 107-114. (2018).
5. Dirany A，Sirés I，Oturan N，et al．. Electrochemical abatement of the antibiotic sulfamethoxazole from water. Chemosphere. 81(5): 594-602 (2010).
6. Hu, Y., Yu, F., Bai, Z., Wang, Y., Zhang, H., Gao, X., Wang, Y., Li, X. Preparation of Fe-loaded needle coke particle electrodes and utilisation in three-dimensional electro-Fenton oxidation of coking wastewater. Chemosphere 136544.(2022).
7. Zhang, Y. , Chen, Z. , Wu, P. , Duan, Y. , Zhou, L. , & Lai, Y. , et al. Three-dimensional heterogeneous electro-fenton system with a novel catalytic particle electrode for bisphenol a removal. Journal of Hazardous Materials. 393: 120448-120453 (2019).
8. Sun Yongjun, Peng Li, Huaili Zheng, Chun Zhao, Xuefeng Xiao, & Yanhua Xu.Electrochemical treatment of chloramphenicol using ti-sn/γ-al2o3 particle electrodes with a three-dimensional reactor. Chemical Engineering Journal. 308, 1233-1242 (2017).
9. Li, H. , Yang, H. , Cheng, J. , Hu, C. , Yang, Z. , & Wu, C. Three-dimensional particle electrode system treatment of organic wastewater: a general review based on patents. Journal of cleaner production(Jul.25), 308. 127324.1-127324.26. ISSN:0959-6526 (2021)..
10. J.N. Sahu, J. Acharya, B.C. Meikap. Optimization of production conditions for activated carbons from Tamarind wood by zinc chloride using response surface methodology, Bioresour. Technol. 6 1974-1982. (2010)
11. Saeed Sadeghpour Galooyak, B. Dabir. Three-factor response surface optimization of nano-emulsion formation using a microfluidizer, J. Food Sci. Technol. 52: 2558-2571 (2015).
